# The Assessment of Autoimmunological Status and Prevalence of Different Forms of Celiac Disease among Children with Type 1 Diabetes Mellitus and Celiac Disease

**DOI:** 10.1155/2008/285989

**Published:** 2008-04-16

**Authors:** Grazyna Deja, Anna Myrda, Przemyslawa Jarosz-Chobot, Urszula Siekiera

**Affiliations:** ^1^Department of Pediatrics, Endocrinology and Diabetes, Medical University of Silesia, 40-752 Katowice, Poland; ^2^Schwarzwald-Baar Klinikum, 78011 Villingen-Schwenningen, Germany; ^3^Blood Center, 40-074 Katowice, Poland

## Abstract

This study aims to assess the autoimmunological status and forms of celiac disease (CD) among children with type 1 diabetes mellitus (T1DM). The study group comprises 27 patients at the mean age of 12.30 years (±SD 3.12). The measurement of the level of diabetes-specific antibodies and organ-specific antibodies was gained at the T1DM-onset and repeated annually. The following risk factors influencing time of CD diagnosis were analyzed: age, sex, T1DM duration, autoantibodies, and HLA-haplotype. The prevalence of antibodies was GADA-74%, IAA-63%, IA2A-67%, ATA-11%, and ATG-4%. The intestinal biopsy revealed in 19% no changes and in 77% stage 3 (Marsh scale). In most cases, no clinical manifestation of CD was observed. The diagnosis of Hashimoto's disease was made twice. The negative correlation between the age at T1DM-onset and the interval between onset of T1DM and CD (*r* = −0.35, *p* < .05) was noted. The high-comorbidity ratio of CD and thyroiditis with T1DM demands regular screening tests especially in the first years after T1DM-onset.

## 1. INTRODUCTION

Due to a common genetic background and interaction between environmental and immunological factors, patients with type 1 diabetes mellitus (T1DM) are at a high risk of having other autoimmunological diseases. The autoimmune polyendocrine syndromes type 1 (APS-1) and type 2 (APS-2) are rare but most dramatic. APS-1 is caused by a single mutation in the AIRE gene (transcriptional factor) as opposite to APS-2, which is a “complex” genetic disorder with strong HLA-genes association (haplotype DQA1∗0501, DQB1∗0201, DRB1∗0301) [1]. Single autoimmune diseases like celiac disease (CD) or thyroid gland disease are more common among patients with T1DM. They are also connected with above-mentioned predisposing HLA-genes. In the course of T1DM, no clinical manifestation of both coexisting disorders is noted in most cases [2, 3]. That is why regular investigations of the autoimmunological status should be provided. Severe complications of untreated celiac disease necessitate an early diagnosis and the prompt introduction of a gluten-free diet. Undiagnosed thyroid gland disease that remains untreated can lead to subclinical or clinical obvious hypothyroidism.

At the time of the diagnosis of diabetes, various antibodies are evaluated. To make a statement of autoimmune background of the disease, the diabetes-associated antibodies are detected. Blood samples are screened for islet cell antibodies, insulin autoantibodies, GAD antibodies, and anti-IA-2 antibodies [4, 5]. Additionally, screening for celiac disease and autoimmune thyroiditis is usually performed. The presence of humoral immune markers allows an immediate medical intervention, but their absence should not mislead a physician, since there is a possibility of developing these disorders some years after diabetes onset [5].

The purpose of our study was to assess retrospectively the autoimmunological status from the time of the first diabetes presentation until celiac disease was revealed and assess the risk factors of influencing the time of the CD diagnosis. The next objective of the study was to evaluate the stages of celiac disease most commonly appearing among children with type 1 diabetes mellitus.

## 2. MATERIALS AND METHODS

The study group comprises 27 diabetic children (14 girls, 13 boys) at the mean age of 12.3 years (±3.12) with diagnosed celiac disease. They were selected from about 450 new patients with T1DM. The retrospective analysis concerns patients' data collected in the years 2001–2006. All patients taken into consideration were hospitalized in the Department of Pediatrics, Endocrinology and Diabetes in Katowice at the time of the first diabetes presentation. Patients were regularly observed in outpatient diabetes care at least till CD was revealed. The diagnosis of T1DM was made according to WHO and ISPAD criteria [4, 5].

The measurement of the level of disease-associated autoantibodies to glutamic acid decarboxylase (GADA), protein-tyrosine phosphatase (IA2A), insulin (IAA) was gained at the time of T1DM onset. The serum samples were drawn before the initiation of insulin treatment. Samples for organ-specific antibodies: tissue transglutaminase (tTG), thyroperoxidase (ATA), and thyroglobuline (ATG) as well as thyroid hormones (TSH, fT4) were also obtained from diabetes-onset patients. In all children, serum immunoglobulin A (IgA) concentration was determined to exclude IgA deficiency and a subsequent false interpretation of received results of IgA tTG. The control measurements of tTG, ATG, ATA, and thyroid hormones were repeated annually. The titer of GADA, IA2A, and IAA was gauged by using the radioimmune assay technique (RIA-CIS Bio International) in conformity with the protocols included in laboratory original kits. The measurement ranges were as follows: for GADA 0–300 U/mL, for IA2A 0–50 U/mL, and for IAA 0–100%. The results for GADA and IA2A above 0.75 U/mL and for IAA above 7% were considered positive. The CD-associated IgA tTG were detected by the ELISA method. The level of thyroid hormones and thyroid antibodies was estimated by the chemiluminescence method (DPC, USA). Normal ranges for fT4 for children under the age of 12 are 0.65–2.3 ng/dl and for older ones are 0.8–1.9 ng/dl. TSH was assessed according to age-adequate normal levels. The concentration of ATA > 35 IU/mL and of ATG > 40 IU/mL was considered to be positive. IgA concentration was assessed by immunoturbidimetric measurements (Dade Behring, Germany). IgA deficiency was defined as an IgA below-normal level adequate for age.

If autoimmunity markers were present, the ultrasound examination of thyroid gland or the small bowel biopsy was ordered. Intestinal mucosa specimens were obtained during peroral endoscopy and afterwards examined by a pathologist. Changes in mucosa were assessed by employing the Marsh stage. Type 1 (infiltrative lesion) is characterized by normal structure of mucosa with presence of intraepithelial lymphocytes infiltration. Type 2 (hyperplastic lesion) comprises type 1-mentioned changes and additionally enlarged jejunum crypts. Type 3 (destructive lesion) is divided into 3 groups. The characteristics of all of them include inflammatory infiltration, hyperplastic crypts, and the villous atrophy, which in each group is of a different degree. In type 3a partial, in type 3b subtotal, and in type 3c, total destruction of villi is observed. Type 4 (hypoplastic lesion) comprises total villous atrophy without inflammatory infiltration and the normal architecture of the crypts.

HLA tests were performed in the Regional Blood Centre in Katowice. HLA class II alleles were typed by using the polymerase chain reaction—the single strand polymorphism method (PCR-SSP). Allele-specific tests One Lambda (Cytogen, USA) were employed for HLA-DRB1∗ and DQB1∗ typing. For genetic assays, human leukocytes were used. The PCR program was performed according to the protocol described earlier [6].

The statistical analysis was carried out with the application SigmaStat. To estimate the risk factors influencing the time of CD diagnosis among children with T1DM, the following parameters were analyzed: age at T1DM onset, gender, T1DM duration, presence of autoantibodies, and HLA haplotype. The normality of the distribution of the continuous variables was evaluated by using the Shapiro-Wilk test. The correlation between analyzed parameters was calculated by using Pearson correlation. The results with the *p* < .05 were considered to be statistically significant.

## 3. RESULTS

**Figure 1:**
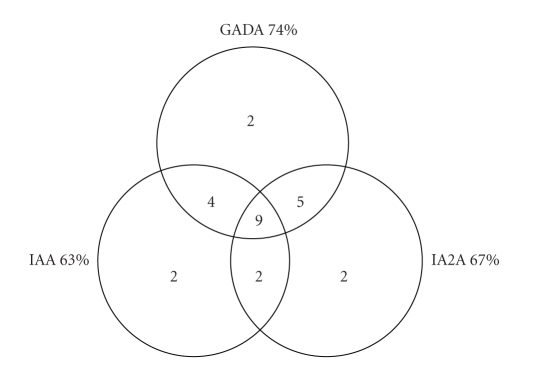
Diabetes-associated autoantibodies in children at the time of T1DM onset.

The mean age at T1DM diagnosis was 7.39 years (±SD 3.12), and the mean age of CD diagnosis was 8.43 years (±3.69). The prevalence of antibodies was as follows: GADA 74%, IAA 63%, and IA2A 67%. One third (33%) of the probands was tested positive for all three autoantibodies. In 11 (41%) cases, the presence of two autoantibodies was identified. In 5 cases GADA+IA2A, in 4 cases GADA+IAA, and in 2 cases IAA+IA2A were detected. Only in one case no autoantibodies were detected (Figure 1).

Thyroid autoimmunity markers were present in 3 (11%) children: 3 (11%) ATA and 1 (4%) ATG. All of them were euthyroid. In 2 (7%) children, abnormal ultrasound thyroid gland image was observed, suggesting Hashimoto's disease. In these children, therapy with L-thyroxine was introduced. A total of 27 intestinal biopsies were performed. The biopsies revealed: 5 (19%) x no changes (latent form), 1 (4%) x type 1, 6 (22%) x type 3a, 6 (22%) x type 3b, and 9 (33%) x type 3c. In the case of 1 girl, CD 3a was present 3 years before she became diabetic. Only once were the T1DM and CD diagnosis made at the same time. In most cases no clinical manifestation was observed.

The HLA haplotypes were obtained from 24 diabetic patients. Genetic typing results of HLA-DRB1 and DQB1 on the low-resolution level showed predominance of the following haplotypes: DRB1∗04-DQB1∗03 and DRB1∗03-DQB1∗02. These T1DM at-risk and CD at-risk haplotypes were found in 8 cases (33%) and in 14 cases (59%), respectively. In 6 patients (25%), both predisposed haplotypes were detected. Two patients expressed DRB1∗03-DQB1∗02 homozygosity. There were no significant differences in frequency of haplotype in relation to sex, age at onset, diabetes duration, or celiac disease form (see Table 1).

**Figure 2:**
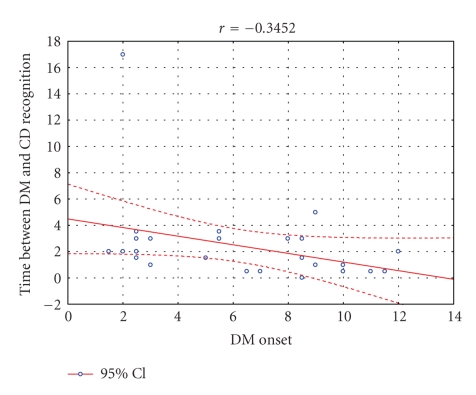
Negative correlation between age at T1DM-onset and the interval between onset of T1DM and CD (*r* = −0.35, *p* < .05).

The analysis of the CD risk factors revealed negative correlation between the interval between onset of T1DM-CD and age at T1DM onset (*r* = −0.35, *p* < .05). There were no other statistically significant correlations between factors taken into consideration (age, gender, T1DM duration, presence of autoantibodies, and HLA-haplotypes) (see Figure 2).

## 4. DISCUSSION

Our study supports the theory of elevated frequency rate of appearance of other autoimmunological diseases in the course of T1DM. The risk of celiac autoimmunity in certain populations is as high as 1 in 100 [7, 8]. The prevalence of celiac disease in children from our diabetes care is at about 6%, what is similar to other studies [8–11]. Moreover, 11% of studied children had thyroiditis markers. The level of coexistence for thyroid- and CD-associated antibodies in diabetic children was described in the previous studies at about 10% [12] and at about 6% [13].

Celiac disease more and more often appears either as symptomatic nonclassic or as a symptom-free disease [2, 3]. Asymptomatic CD can occur in a silent or latent form. An equally predictive value characterizes most commonly provided serological tests for CD. These are the IgA endomysial and/or IgA tissue transglutaminase antibodies. In our practice, we employ IgA tTG antibodies, which is well in accordance with the suggestions NASPGHAN guidelines from 2005 [14]. Also Kordonouri et al. indicate a positive predictive value and higher than IgA EMA antibodies sensitivity in patients with T1DM and silent CD [15].

All our patients with negative screening results at T1DM onset were diagnosed to develop CD within 4 years. In other investigations, the interval was as follows: 4 years [11] and 2 years [16]. These results confirm the importance of regular, annually performed control tests, especially in the first years after becoming diabetic. The asymptomatic course and long exposition to gluten are probably the reason for such a high frequency of Marsh 3c in our study group, as well as the personal sensitivity and predispositions. We have only 1 girl with CD diagnosed before T1DM onset. It confirms other authors' observations that CD rarely precedes the presentation of T1DM [11, 17].

We found negative correlation between age at T1DM onset and the interval between onset of T1DM and CD. In the case of other risk factors, such as age, gender, and T1DM duration we did not observe statistically significant correlations. The opinions of other authors about the risk factors of developing CD are inconsistent; some authors indicate females to be at higher risk of having both diseases [2, 18].

The majority of cases of CD coexisting with T1DM are symptom-free at CD-diagnosis [2, 3]. Often no signs of overt malnutrition, no gastrointestinal symptoms, no dermatological problems, or recurrent hypoglycemia episodes are noted [2]. Also, the most of our patients did not present any symptoms of digestive system and no symptoms of malnutrition and height deficiency. It is in accordance with the results of the longitudinal observational study published by Valletta et al. [19] which shows that at diagnosis of CD screened subjects with T1DM had only minor signs of malnutrition (measured by weight, height and BMI). CD in our subjects was confirmed by a small bowel biopsy and a histological examination of intestinal specimens, which remains the gold standard method for the diagnosis [14]. In every positive subject with diagnosed CD, a gluten-free diet was initiated. In children with a diagnosed latent form of CD, serological tests and further clinical investigations are needed since the overlap either into a clinically overt or silent form of CD is possible. Preventional treatment in the form of a gluten-free diet in children with T1DM and silent CD also appears for us to be justifiable [11, 20].

Autoimmune thyroiditis and T1DM can also frequently coexist and take an unhomogeneous course. It often stays undiagnosed because of lack of symptoms [21, 22]. In spite of the presence of organ specific antibodies, such as thyroperoxidase and thyroglobuline, in about 20–30% patients with diabetes, the rate of hypothyroidism is lower (5–10%), and changes in the ultrasound image of thyroid gland are not frequently observed. Polish studies also confirm the high comorbidity of prevalence of autoantibodies and thyroid disorders among patients with T1DM [22, 23]. In our group of patients with T1DM and CD, the analysis regarding thyroid autoimmunity revealed 3 (11%) patients with positive results. Maybe such a small number of positive results is caused by our study inclusion criteria (having both disorders: T1DM and CD). The second possible explanation of this finding is the fact that children included to the study were rather young (only 3 of them had the T1DM-onset above 10 years) and thyroid disorders usually developed in older age. In two (8%) of 27 children, the diagnosis of Hashimoto's disease was made on the basis of the serum findings and ultrasound images. All these patients had negative screening results at T1DM onset and also were euthyroid and asymptomatic at the time of diagnosis. Data coming from literature pointed that the asymptomatic course of the disease is rather typical and indicated the benefits of annual TSH screening to hypothyroidism development [24].

Similar to previously investigated Caucasian populations, also in our study haplotypes DRB1∗04-DQB1∗03 and DRB1∗03-DQB1∗02 revealed the predominance, since these are most strongly connected with T1DM haplotypes [25]. Nevertheless, in the studied group of children with coexisting CD and T1DM, we observed more frequent presence of allele DQB1∗02 compared with DQB1∗03 (59% versus 33%, resp.). Such predominance of allele DQB1∗02 has not been noted in our previous study concerning a genetic susceptibility to T1DM in the same population (65% versus 62%, resp.) [26]. It confirms earlier observations that the most strongly connected with CD allele is DQB1∗02, which is present in about 90% of CD patients [27, 28]. The high importance of the allele DQB1∗02 in developing CD especially confirms the Bao study [25]. In this study it was shown that approximately 1 out of 3 patients with T1DM, who are DQB1∗02 homozygous, expresses tTG-antibodies, and half of them have celiac disease on biopsy. In our study group, we had only 2 children expressing DQB1∗02 homozygosity. In both cases, the biopsy was positive: stage 3a and 3c were recognized.

Diabetes-associated autoantibodies were found in most subjects at the time of T1DM presentation. Our group of patients is positive to all antibodies: GADA, IA2A, and IAA; (33%) seems to be rather large in comparison to a group presented for example by Sabbah et al. (2, 1%) [29]. The explanation for such a large group of children positive to multiple autoantibodies could be the fact of having additionally 2 or 3 autoimmune disorders and an individually increased tendency to autoimmunization. Furthermore, we noted the GADA dominance in comparison to other markers. Probably these both facts can result from more frequent presence of the allele DRB1∗03 in the studied group. The allele DRB1∗03 is known as the most classic haplotype connected with a different form of autoimmunization and a generally increased tendency to produce autoantibodies. GADA is also detected more frequently and in higher titer in subjects with haplotype DRB1∗03 [30, 31]. But because of a rather small number of children comprising our study group, it is not possible to make an obvious conclusion based on statistically significant calculations. It indeed makes a limitation of our study and indicates the necessity of continuation of our observation to obtain results of more subjects with T1DM and CD.

## 5. CONCLUSION

In conclusion, the high-comorbidity ratio of CD and thyroiditis in the course of T1DM, especially in a latent and silent form, demand regular screening tests, especially in the first years after T1DM-onset. The increase in the number of patients with T1DM and CD and longer observations are needed to assess the risk factors influencing the time of CD onset. It is especially important in the context that the newest data coming from study performed in 7 big European Diabetic Centers confirmed rapidly the increasing trend of prevalence of both disorders [32] and because of only a few data on the predictive value and risk factors of CD in T1DM populations published so far [33].

## Figures and Tables

**Table 1:** Study group characteristics.

	Gender (female = 1)	Age at T1DM onset	GADA	IAA	IA2A	ATA	ATG	Biopsy	HLA
1	0	11.5	−	+	+	+	+	3b	DRB1∗03 DQB1∗02 DRB1∗04 DQB1∗03
2	1	2.50	+	+	+	−	−	3b	DRB1 07 DQB1 02 DRB1∗04 DQB1∗03
3	1	5.50	+	−	+	+	−	3a	DRB1∗03 DQB1∗02 DRB1∗16 DQB1∗05
4	0	5.00	−	+	−	−	−	0	DRB1∗03 DQB1∗02 DRB1∗04 DQB1∗03
5	0	10.00	+	+	−	−	−	1	—
6	1	5.50	+	+	−	−	−	0	—
7	0	2.50	+	+	+	−	−	3c	DRB1∗03 DQB1∗03 DRB1∗04 DQB1∗02
8	1	5.50	−	+	+	−	−	3a	DRB1 07 DQB1 03 DRB1 16 DQB1 06
9	1	7.00	+	−	−	−	−	3c	DRB1∗03 DQB1∗02 DRB1∗04 DQB1∗03
10	0	3.00	+	+	+	−	−	0	DRB1∗03 DQB1∗02 DRB1∗04 DQB1∗03
11	1	2.00	+	+	+	−	−	3a	DRB1 01 DQB1 02 DRB1 16 DQB1 03
12	1	3.00	−	−	+	−	−	0	—
13	1	8.00	+	+	−	−	−	3c	DRB1∗03 DQB1∗03 DRB1∗04 DQB1∗02
14	0	1.50	+	+	+	−	−	3b	DRB1∗04-DQB1∗03 DRB1 07 DQB1 02
15	0	2.00	−	+	−	−	−	3c	DRB1∗03-DQB1∗02 DRB1 06 DQB1 05
16	1	2.50	+	−	+	−	−	0	DRB1∗03 DQB1∗02 DRB1∗09 DQB1∗02
17	1	9.00	−	−	−	−	−	3b	DRB1∗03 DQB1∗02 DRB1∗04 DQB1∗03
18	0	5.00	+	−	+	−	−	3c	DRB1 01 DQB1 04 DRB1∗03 DQB1∗02
19	1	8.50	+	−	+	+	−	3b	DRB1∗011DQB1∗03 DRB1∗011DQB1∗02
20	0	10.00	−	−	+	−	−	3a	DRB1∗03 DQB1∗02 DRB1∗07 DQB1∗03
21	1	6.50	+	+	+	−	−	3c	DRB1∗03 DQB1∗02 DRB1∗13 DQB1∗03
22	1	2.50	+	+	+	−	−	3c	DRB1∗03 DQB1∗03 DRB1∗04 DQB1∗02
23	0	8.50	+	−	+	−	−	3a	DRB1∗03 DQB1∗03 DRB1∗04 DQB1∗02
24	0	11.00	+	+	−	−	−	3a	DRB1∗03 DQB1∗02 DRB1∗03 DQB1∗02
25	0	9.00	+	+	+	−	−	3c	DRB1∗03 DQB1∗02 DRB1∗03 DQB1∗02
26	0	8.50	+	+	+	−	−	3b	DRB1∗07 DQB1∗03 DRB1∗15 DQB1∗06
27	1	12.00	+	−	−	−	−	3c	DRB1∗03 DQB1∗03 DRB1∗04 DQB1∗02
